# Infiltration of micro-fragmented adipose tissue under local anesthesia for knee osteoarthritis treatment is a safe procedure: A case series

**DOI:** 10.1016/j.clinsp.2024.100527

**Published:** 2024-12-03

**Authors:** Bruno Butturi Varone, Daniel Peixoto Leal, Daniel Duarte Perini, Vitor Penteado Figueiredo Pagotto, Riccardo Gomes Gobbi, Marco Kawwamura Demange

**Affiliations:** Hospital das Clínicas da Faculdade de Medicina da Universidade de São Paulo (HC-FMUSP), São Paulo, SP, Brazil

**Keywords:** Knee osteoarthritis, Mesenchymal stem cell, Micro-fragmented adipose tissue, Adipose stem cells, Adult stem cells

## Abstract

**Introduction:**

Knee osteoarthritis is a debilitating disease that has been increasing in prevalence, especially due to the aging population and rising incidence of obesity. Biological therapies (orthobiologics) have emerged, demonstrating efficacy in improving functional scores, including the injection of microfragmented Adipose Tissue (mFAT). The purpose of this study is to evaluate the safety of adipose tissue harvesting and mFAT injection under local anesthesia.

**Materials/methods:**

A case series involving 34 patients who underwent adipose tissue collection from the abdomen under local anesthesia, without sedation, in a sterile environment, and who received the infiltration of microfragmented adipose tissue after single-time preparation, using a fat washing and microfragmentation kit.

**Results:**

Evaluation was performed at the time of the procedure and after 7 days of follow-up. Only ecchymosis (76.5 %) and mild discomfort at the harvested site (70.6 %), as well as mild discomfort at the infiltrated knee (61.8 %), were identified. No major adverse events were identified.

**Conclusion:**

The harvesting and injection of mFAT under local anesthesia is safe, with no major adverse events identified during this procedure.

## Highlights

• Harvesting abdominal fat for mFAt knee injection is a safe procedure.
• Minor events, such as ecchymosis, mild abdominal discomfort, and mild discomfort at the infiltrated knee are common.
• The procedure can be done under local anesthesia, without sedation after single-time preparation.

## Introduction

Knee Osteoarthritis (OA) is one of the most prevalent joint diseases worldwide,^1^ affecting over 13 % of men and 10 % of women over 60-y60 years.^2^ It is estimated that this prevalence will increase with the aging population and the prevalence of obesity. As an inflammatory/degenerative joint disease, OA causes significant pain, limits joint function, and significantly affects the patient's life quality.^2^

Evidence suggests that OA is an inflammatory condition, characterized by joint synovitis with increased mononuclear cells and pro-inflammatory mediators, positive regulation of aggrecans and collagenases.^3^ Clinical and epidemiological studies have shown that inflammation is a common feature in the knee joint of individuals diagnosed with OA and is associated with the progression of lesions and degeneration of articular cartilage.^4^, ^5^, ^6^ These observations suggest that suppressing the inflammatory process using corticosteroids may reduce the progression of knee osteoarthritis. The effectiveness of corticosteroid use in joint infiltrations for OA treatment has been demonstrated in animal models,^7^ as well as being established and widely used in clinical practice for knee OA treatment by orthopedic surgeons.^8^ However, the effectiveness of this therapy has a short duration, which has prompted research into new intra-articular infiltration therapies.

More recently, biological and regenerative therapies have begun to provide new perspectives within the field of orthopedics. These therapies can expand non-surgical or minimally invasive treatment options available for patients with early OA.^9^ Orthobiologics are naturally found in the human body. The most studied orthobiologics currently are Platelet-Rich Plasma (PRP), hyaluronic acid, bone marrow aspirate, and microfragmented Adipose Tissue (mFAT).

The aim of this study is to evaluate the safety of performing the autologous fat graft harvesting procedure, its preparation, and intra-articular infiltration under local anesthesia.

## Material and methods

This is a case series study with 34 patients, performed in a single center, in the period from November 2021 to December 2022.

Patients with symptomatic OA of the knee (Kellgren-Lawrence Grade 2‒4) were eligible for inclusion if aged 40‒80 years. Patients were excluded from the study if they had varus or valgus malalignment of the knee exceeding 10°, rheumatologic arthritis, or BMI above 40 kg/m^2^. Patients were informed of the study verbally and in writing before written consent was obtained.

Patients were treated with a 10 mL intra-articular injection with autologous, microfragmented adipose tissue prepared using the Lipogems system.

### Patient set-up

Adipose tissue was collected under sterile conditions in a surgical center under local anesthesia, without sedation. In all patients, intravenous access was obtained for the administration of cefazolin 2 g as antibiotic prophylaxis, sodium dipyrone 1 g, and dimenhydrate 50 mg.

Adipose Tissue (AT) harvest site was performed in the lower abdomen. The portals were marked above the inguinal line, on each side of the abdomen.

The skin anesthesia of 1 mL of 2 % lidocaine was applied in the portal location. After the anesthetic latency time, a small incision of approximately 4 mm was made with an 11-blade scalpel on each side of the abdomen. For harvesting the adipose tissue, a two staged intumescent technique was performed.

The first step consists in the insertion of a 19 G cannula through the portal for subcutaneous tissue dispersion. The anesthetic solution consisted of 20 mL of 2 % lidocaine, 20 mL of 0.5 % bupivacaine, 1 mL of 1 mg/mL adrenaline, and 250 mL of 0.9 % saline. Totaling a volume of 291 mL. We used 120 mL of this solution in each hemi-abdomen, the remaining 51 mL was reserved to be used in case we needed more anesthesia during the harvesting. Both cannulas were provided by Lipogems® kit.

### Adipose tissue harvesting

A vaclock syringe was connected to the cannula, to ensure pressure in the system and an optimal fat tissue harvest.

Adipose tissue was harvested in a homogeneous way to avoid cosmetic issues. Pinching the abdomen to evaluate the amount of remaining subcutaneous tissue in each area is a reliable way to avoid any cosmetic changes.

We planned to harvest 120 mL of adipose tissue which would lead to approximately 20 mL of mFAT. Our goal was to inject 10 mL of mFAT on each knee. After the procedure, skin portals were closed with Nylon 5.0 sutures, and Adipose tissue was processed using Lipogems®, a single-use and disposable kit.

### Adipose tissue processing

Adipose tissue was processed using Lipogems®, a single-use and disposable kit that through mild mechanical forces, washing, and reduction filters eliminates oil from ruptured adipocytes and red blood cells present in the adipose tissue aspirate.

**Fig. 1:**
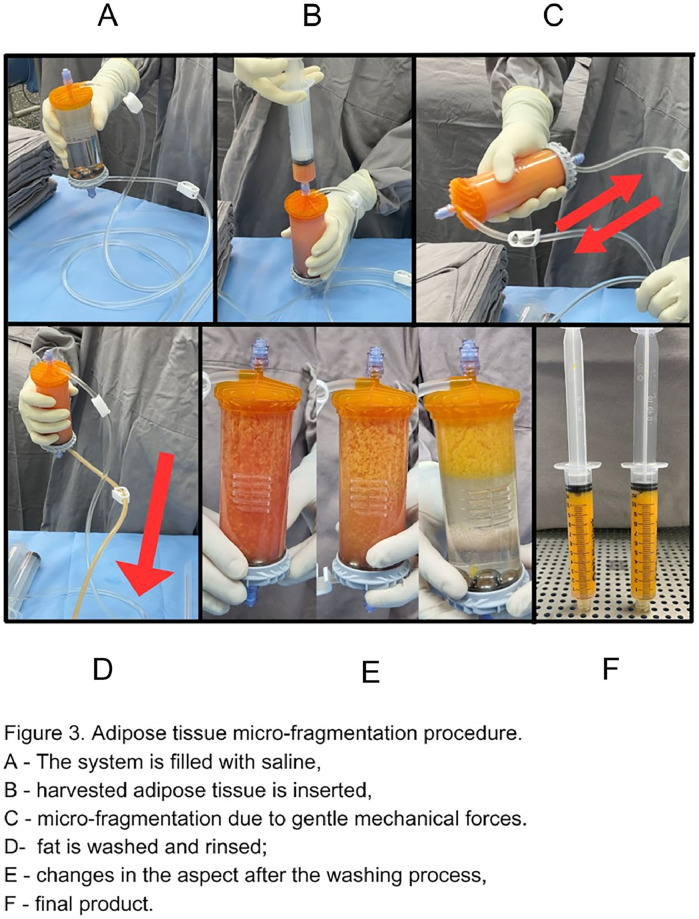
Adipose tissue micro-fragmentation procedure.

The harvested adipose tissue was inserted into the system, which was already prefilled with saline. After that, mechanical dissociation was obtained by gently shaking the system. Stainless steel marbles inside provide additional mechanical fragmentation. Oil residues and blood components are washed out by gravity counter-flow of saline solution, this procedure was repeated until the solution in the divide appears clear and the lipoaspirate is yellow. Micro-fragmented adipose tissue migrates to the top of the device. Finally, the device is turned upside down by 180°, with the fat tissue product now facing a narrower size reduction filter. MFAT is then removed from the device and reserved in 10 mL syringes (Fig. 1).

### Ultrasound-guided intra-articular infiltration

knees were prepared with 2 % degerming chlorhexidine gluconate, then cleaned with an alcoholic chlorhexidine solution, in addition to placing sterile drapes. The area was anesthetized with a 1 mL anesthetic button and deep tissues were anesthetized with an additional 1 mL of 2 % lidocaine.

A 16 G Jelco needle was inserted into the suprapatellar bursa under the guidance of a Logiq E GE Healthcare® ultrasound device and a 12 MHz linear probe to ensure that the infiltration of the product was articular. If there was a joint effusion, the excess fluid was drained. The microfragmented fat tissue was then infiltrated. The orthopedic surgeon who performed the joint infiltration has experience in the area, and the use of ultrasound during infiltration increases the precision and degree of certainty that the product was delivered to the joint cavity.

### Post-operative care

We planned a same-day discharge for all patients. Any delay in the discharge would be recorded. Stitches were removed on a seven-day follow-up. Patients were instructed to avoid physical activities or more intense efforts for one week. The home prescription consisted of dipyrone sodium in cases of mild pain and tramadol in cases of more intense pain. Hirudoid® (mucopolysaccharide polysulfate) was recommended in its topical gel form to minimize bruising. Furthermore, cryotherapy was indicated to control pain, edema, and bruising.

The patient was evaluated within seven days of the procedure to evaluate adverse effects due to adipose tissue harvesting and the bilateral knee injection. The patient was instructed to report any discomfort or pain to the medical team.

## Results

34 patients with knee osteoarthritis were selected.

The average age of participants was 61 ± 9.2 years old, and the average BMI was 26.8 ± 4.5 kg/m^2^.

Twenty-eight (82.4 %) were female.

17 patients (50 % of the total) had comorbidities such as diabetes mellitus and/or systemic arterial hypertension.

Regarding bilaterally, 25 patients had bilateral osteoarthritis, and 9 had unilateral. The probable cause of osteoarthritis is specified in Table 1.

**Table 1:** Perioperative adverse effects.

Perioperative adverse effect (AE), n (%)	MFAT (*n* = 34)
No	30 (88.2)
Yes	4 (11.8)
Adverse effects, n (%)	
Knee pain	2 (5.8)
Discharge postponed	0
Pain during the harvesting	2 (5.8)
Harvested adipose tissue in mL (SD)	117.9 ± 43.2
MFAT obtained in mL (SD)	28.1 ± 8.3
Adverse effect within 7 days, n (%)	
Ecchymosis	26 (76.5)
Abdominal discomfort	24 (70.6)
Knee discomfort	21 (61.8)
Cosmetic changes	0

Regarding the frequency of OA degrees according to the Kellgren Lawrence classification, considering a total of 59 infiltrated knees. 17 knees were classified as Grade 2, 30 knees as Grade 3, and 12 were classified as Grade 4.

Finally, while identifying the cause of knee osteoarthritis, it was found that 28 participants had idiopathic osteoarthritis. In two patients, the cause was identified as being related to meniscal injury, followed by meniscectomy. Three patients had a history of anterior cruciate ligament reconstruction, and one patient had a history of patellar instability.

Results are shown in Table 2.

**Table 2:** Patient characteristics.

Baseline patient characteristics	MFAT (34 patients)
Age, mean ± SD, y	61 ± 9.2
Gender, n (%)	
Male	6 (17.6)
Female	28 (82.4)
BMI, mean ± SD, m kg/m^2^	26.8 ± 4.5
Laterality, n (%)	34 patients
Left	3 (8.8)
Right	6 (17.6)
Bilateral	25 (73.5)
Kellgren Lawrence, n (%)	59 knees
KL 2	17 (28.8)
KL 3	30 (50.8)
KL 4	12 (20.3)
Comorbiditiesª, n (%)	
Yes	17 (50)
Causas da Osteoartrite, n (%)	
Unknown	28 (82.4)
Prior meniscectomy	2 (5.9)
Prior ACL reconstruction	3 (8.8)
Prior patellar instability	1 (2.9)

ª Hypertension and Diabetes were considered as systemic comorbidities.

Among the 34 cases performed, we only identified adverse effects in 4 cases.

Two patients (5.8 %) experienced knee pain while performing the interarticular knee injection with mFAT, while 2 patients (5.8 %) experienced pain during the harvest of the adipose tissue. All patients were discharged on the same day, as expected.

The average volume of adipose tissue harvested was 117.9 ± 43.2, and after the microfragmentation process was 28.1 ± 8.3.

Within 7 days, we observed that 26 patients (76.5 %) presented ecchymosis, 24 patients (70.6 %) presented abdominal discomfort, and 21 patients (61.8 %) reported knee discomfort in this period. No patient complained about cosmetic changes within 7 days (Table 1).

## Discussion

In our case series, 34 cases of adipose tissue graft harvesting were performed in the abdominal region of patients, followed by preparation of mFAT and subsequent infiltration into the affected knee with moderate to severe osteoarthritis. To our knowledge, this is the first study to assess the safety of this procedure under local anesthesia.

The mean age of 61±9.2 years old, the higher prevalence of Female patients enrolled (82.4 %) and the elevated mean BMI (26.8 ± 4.5) represent the population most affected by knee osteoarthritis according to the literature.^10^

Regarding the severity of the disease, we found a high prevalence of bilateral disease (73.5 %), and also a high percentage of knees with advanced osteoarthritis (KL 3 or 4). Considering the Kellgren Lawrence classification, moderate to severe disease accounts for 71.1 % of total knees injected. The severity of the pooled cases can possibly limit the efficacy of mFAT injection, but does not impact the safety assessment of the procedure.

The presence of comorbidity in 50.0 % of cases is typically found in patients with knee osteoarthritis. This high prevalence rate is in line with the literature which, in addition to identifying similar prevalence rates to ours, also suggests that some types of comorbidities may be related to a worse prognosis of joint disease.^11^

Concerning the etiology of the KOA, 28 patients were classified as having an idiopathic cause. The large number of cases without a defined etiology probably arises from the chronicity of the disease, associated with difficult access to healthcare. Possibly associated with neglected chondral or meniscal injuries that progressed unfavorably until being referred to a tertiary service.

During the procedure, an average volume of 117.9 ± 43.2 mL of adipose tissue was collected, with a microfragmented final volume of 28.1 ± 8.3 mL obtained. According to recent literature, 8‒10 mL is recommended for a bigger joint such as the knee. Thus, with the volume of adipose harvested, we were easily able to process and inject bilateral knees.

Among the 34 cases, only 2 patients experienced significant discomfort at the graft harvesting site, which was managed by reapplication of local anesthetic (Klein's solution) and intravenous analgesia with a common analgesic (1 g dipyrone). Another 2 patients experienced discomfort during joint infiltration, which was also managed by the application of local anesthetic, without compromising the procedure. In most of the studies with mFAT, the harvesting is performed under general anesthesia since an additional procedure is also performed such as osteotomies, arthroscopic debridement or meniscectomies. Different from previous studies, this is a local anesthesia procedure only. Having this challenge in mind, our initial local anesthesia volume prepared was 291 mL (250 mL of saline 0.9 %, 20 mL of bupivacaine and 20 mL of lidocaine), but in the initial subcutaneous tissue infiltration with anesthetics solution only 250 mL was injected through the 19 G Canula. Therefore, we had available 41 mL for a complimentary anesthetics injection as needed.

During the first week postoperative follow-up, mild ecchymosis was observed in the abdomen of 26 patients, mild abdominal discomfort in 24 patients, and knee discomfort (pain and sensation of heaviness) in 21 out of 34 patients. No major adverse effects such as bleeding, abdominal cosmetic deformity and deep venous thrombosis were identified.

No postoperative infection was identified, either in the harvested area or in the injected joints.

The results of our case series are consistent with current medical literature, which validates the procedure as safe to be performed by a trained professional in adipose tissue harvesting.^12^, ^13^, ^14^ From previous studies, Hong et al. observed that 25 % of patients reported discomfort during more intense physical activity for one week at the graft harvesting site in the abdomen only.^15^ The high prevalence of ecchymosis and mild abdominal discomfort in both groups was an expected adverse effect. Patients were pre-informed and reassured about this fact. All cases were solved within 1 week period, and no cosmetic changes were evident after that time frame. Our study aligns with literature that highlights low rates of aesthetic complications, with only one previous study reporting a single case of mild aesthetic alteration requiring an additional surgical approach.^13^ A recent study showed safety after 3 years of follow-up in patients who were injected with mFAT and had associated surgical procedures. One case report, however, describes a Baker's cyst inflammation after intraarticular knee injection of mFAT.^16^

Based on the findings, we can infer that autologous fat graft harvesting, preparation, and mFAT infiltration are safe procedures in the treatment of knee osteoarthritis. With adequate procedure sterility and local anesthesia administration, this procedure can be part of the therapeutic arsenal of orthopedic surgeons with proper training in adipose tissue graft harvesting, including in an outpatient setting.

As a limitation of the study, we identified that all procedures were performed by the same orthopedic surgeon with correct training in the technique, which limits the occurrence of adverse effects. Additionally, we observed the limitation of fat harvesting only in the abdominal site, which may be difficult or even impossible in patients with low abdominal adipose deposits. Larger, multicenter studies with harvesting from different sites (medial or lateral thigh, hips, or buttocks) should be conducted to complement the findings of this study.

## Conclusion

The harvesting of autologous adipose tissue graft, its preparation, and intra-articular infiltration under local anesthesia is a safe procedure, and it can be part of the therapeutic arsenal for knee osteoarthritis in an outpatient setting.

## Declaration of competing interest

The authors declare no conflicts of interest.
